# Peak match acceleration demands differentiate between elite youth and professional football players

**DOI:** 10.1371/journal.pone.0277901

**Published:** 2023-03-01

**Authors:** Bradley Thoseby, Andrew D. Govus, Anthea C. Clarke, Kane J. Middleton, Ben J. Dascombe

**Affiliations:** 1 Sport and Exercise Science, School of Allied Health, Human Services and Sport, La Trobe University, Melbourne, Australia; 2 High-Performance Department, Melbourne City Football Club, Melbourne, Australia; 3 Applied Sport Science and Exercise Testing Lab, School of Environmental and Life Sciences, University of Newcastle, Callaghan, Australia; Universidade Federal do Rio Grande do Sul, BRAZIL

## Abstract

Youth footballers need to be developed to meet the technical, tactical, and physical demands of professional level competition, ensuring that the transition between competition levels is successful. To quantify the physical demands, peak match intensities have been measured across football competition tiers, with team formations and tactical approaches shown to influence these physical demands. To date, no research has directly compared the physical demands of elite youth and professional footballers from a single club utilising common formations and tactical approaches. The current study quantified the total match and peak match running demands of youth and professional footballers from a single Australian A-League club. GPS data were collected across a single season from both a professional (n = 19; total observations = 199; mean ± SD; 26.7 ± 4.0 years) and elite youth (n = 21; total observations = 59; 17.9 ± 1.3 years) team. Total match demands and peak match running demands (1–10 min) were quantified for measures of total distance, high-speed distance [>19.8 km·h^-1^] and average acceleration. Linear mixed models and effect sizes identified differences between competition levels. No differences existed between competition levels for any total match physical performance metric. Peak total and high-speed distances demands were similar between competitions for all moving average durations. Interestingly, peak average acceleration demands were lower (SMD = 0.63–0.69) in the youth players across all moving average durations. The data suggest that the development of acceleration and repeat effort capacities is crucial in youth players for them to transition into professional competition.

## Introduction

Football academy systems are an important component of elite football organisations, with their primary aim being to develop youth players for promotion into professional squads [1]. To foster youth player development, longitudinal training plans are implemented to prepare youth players for the physical, technical and tactical demands of professional football [2]. Two primary methods by which successful youth development can be achieved is through either participation in high-level competitive matches, or exposure to training sessions that replicate the demands of the competitive match-play [3]. As such, there is a strong need to understand the physical demands of match-play to proactively prescribe training stimuli that prepare players for the physical demands of competition [4].

Professional football players cover a total distance (TD) of between 9–13 km across a match, of which between 600–1200 m is covered as high-speed distance (HSD; >19.8 km·h^-1^) with players performing between 60–100 accelerations (>2.0 m·s^-2^) [5–7]. Similarly, elite youth players cover a TD of between 9–12 km, of which 300-1100m is completed at high-speeds [8–12]. While HSD volume can distinguish between top- and moderate-class professional football players (based on FIFA rankings) [13], other researchers have reported similar HSD between youth and professional footballers [12]. However, a recent review has highlighted inconsistencies in the speed thresholds used to quantify HSD covered in youth competitions [11], which limits the direct comparisons to professional competition data. Further, a direct comparison of the acceleration profiles of youth and professional players at a single professional Danish club demonstrated that U19 players performed more accelerations than both the U17 and open-age professional cohorts [12]. While total match demands appear similar between youth and professional footballers, it is possible that peak match running demands, i.e. the most physically demanding periods of competition, may differ, with the peak physical match demands being considerably higher than the 90-minute average [4, 14]. As such, research is warranted to explore differences in the peak physical match demands between youth and professional football players. Such information could be used to inform training prescription in programs that aim to develop youth football players into professional players.

With individual training drill durations typically being significantly shorter than match durations (typically 1–10 min in length vs 90 min), quantifying the most physically demanding passages of a match over similar shorter durations ensures that the training stimulus is relevant to the match demands [4]. Recently, such peak demands have been quantified through the application of a moving average for each physical output metric for pre-determined durations (e.g. 1–10 min), from which the maximum recorded value is then extracted [4]. Depending on window length, the peak physical demands have been reported for relative TD (115–205 m·min^-1^), relative HSD (10–65 m·min^-1^) and peak average acceleration (AveAcc; 0.52–0.90 m·s^-2^) across professional football competitions based in Australia and England [4, 15]. Such peak physical outputs are far higher than the 90-minute averages reported in professional competition (TD ~104 m·min^-1^, HSD ~6.5 m·min^-1^) [14]. Hence, the use of full match data to inform training practices will provide an insufficient stimulus to prepare players for the peak physical demands of competition.

Within youth academy systems, the application of peak match running data from professional teams offers value in transitioning players into professional competitions. For example, a five-minute football specific conditioning drill prescribed to youth players can be adapted to replicate the most physically demanding five-minutes of a professional competition match. Additionally, as youth level competitions often accommodate a quota of overage players, (i.e. as a match opportunity for injured professional players returning to full fitness), such youth competitions can be used as a progression toward professional competition. However, there is limited research that has reported on the peak match running demands of youth footballers [16–18], and as such, understanding of whether the peak physical demands of youth competition are sufficient enough to expose players to the demands required at a professional level, is limited. From the available data, the peak match TD and HSD running demands of English U23 development league players [16] appear similar to professional English Championship footballers [15]. Conversely, both the elite Italian (U15-U17) [17] and Spanish youth (U20) [18] have demonstrated lower peak match running demands of TD and HSD than players in the professional Italian and Spanish competitions, respectively [19, 20]. However, such comparisons are limited as they do not share a collective philosophy around team formation and tactical approaches. Importantly, past research has observed that peak match running demands differ with team formations and tactical approaches [6, 15, 20, 21], and therefore controlling these factors is crucial in exploring competition differences in peak physical outputs. Therefore, the current study aimed to quantify and compare the peak match running demands (TD, HSD and AveAcc) of elite youth and professional footballers within a single club that employs consistent team formations and tactical approaches. This direct comparison will provide the best indication of differences between youth and professional footballers and will prove useful in the long-term development of youth athletes to better ensure they are able to meet the peak match running demands required of professional football.

## Materials and methods

An observational design was employed to compare competition differences in peak match running demands across incremental moving average durations of 1–10 minutes in elite youth and professional football players. Data were collected from 21 elite youth (17.9 ± 1.3 yr, 16.1–20.4 years) and 19 professional (mean ± SD, range; age: 26.7 ± 4.0 yr, 20.1–32.0 yr) footballers playing for the same professional club in Australia for every available match across one competitive season of fixtures (number of matches: youth = 8, professional = 23). This equated to a total of 59 and 199 individual match observations for the youth and professional competitions, respectively (professional = 10 ± 7 matches per player, range 1–21; youth = 3 ± 2, 1–7). Goalkeepers were excluded from the analysis. Further, only players who played for more than 70 minutes were included in the data analysis due to majority of peak match demands occurring prior to the 70^th^ minute of match play [22]. Each team utilised the same 4-1-2-3 formation across all matches and while different positional groups have displayed physical demands during match-play [23, 24], players were not sub-divided into positional groups as the small cluster size for each positional group would limit the statistical power. Further, as the primary aim was to determine differences in peak running demands between youth and professional competitions, the amalgamation of all positional groups was deemed appropriate. Additionally, while the direct tactical approaches utilised by each team were unable to be directly quantified, at the club in question, the tactical approaches and football philosophy employed in the youth team were done so under direction from the manager of the professional team to ensure consistency between squads and maximise player development. The protocols used in the current study were submitted to and approved by the La Trobe University Human Research Ethics Committee, with informed written consent obtained from all participants prior to data collection HREC#: 18056. Data were collected in line with contractual agreements set out between the club, players and player guardians (where appropriate).

## Activity profile

Player activity data were collected each match using 18 Hz (10 Hz GNSS) portable GPS units (STATSports, Northern Ireland) positioned under the playing jersey and secured in a custom-made harness between the scapulae. GPS devices were turned on 30 minutes prior to the commencement of a match to allow for satellite acquisition. The GPS units employed are valid and reliable in measuring locomotor speeds of team sport athletes [25]. GPS data were downloaded post-match using proprietary software (STATSports, Northern Ireland), with raw GPS files (inclusive of added time) exported into statistical software (R Studio, v1.2.5033) for further analysis. As per previous methods, the raw exported speed trace was filtered using a 4^th^ order, one-way Butterworth filter with a cut-off frequency of 1 Hz [4]. Individual data points in which running speed exceeded 10 m·s^-1^ and instantaneous acceleration values exceeded 6 m·s^-2^ were classified as technical errors and replaced with zero values.

Based on current athlete monitoring practices, three GPS metrics of running intensity were assessed: TD, HSD (>19.8 km·h^-1^) and AveAcc [26]. Measures of TD and HSD were made relative to playing time (m·min^-1^), with acceleration profiles calculated through the summation of the absolute value of all accelerations and decelerations, averaged over a defined duration to calculate AveAcc (m·s^-2^) [27]. The amalgamation of both accelerations and decelerations into a singular metric, while concealing the underlying mechanism of load has been suggested to better reflect the overall intensity of match play [4]. A moving average technique was applied to all three of the match output variables to calculate the peak match running demands. Ten incremental epochs were used (i.e., 1–10 min), with the maximum value for each epoch, for each variable, recorded and then fitted using a power law curve [4].

### Statistical analysis

R Studio statistical programming software (v1.2.5033, R Core Development Team, Vienna), in conjunction with the *nlme* [28] and *lme4* packages [29], were used to conduct non-linear and linear mixed effects analysis. Non-linear mixed models were used to calculate exponent and slope values for the power law model. Linear mixed models were used to assess differences in competition levels for TD, HSD, and acceleration profiles. In the linear mixed model, fixed effects were included for intensity period [ten levels: 1–10 min] and competition level [two levels: youth and professional]. A random intercept was included for player and an exponential covariance structure, with a nugget effect, to account for temporal autocorrelation between intensity periods.

Raw unit differences between competition level, at each intensity period, were converted to standardised mean differences (SMD) by dividing the mean, raw unit difference by the within-subject standard deviation (SD) attained from the random effects (i.e., the square root of the residual variance term). The magnitude of the within-subject SMD was quantified using the following qualitative descriptors: *trivial* (<0.2), *small* (0.2–0.6), *moderate* (0.6–1.2), *large* (1.2–2.0), *very large* (2.0–4.0) and *extremely large* (>4.0) [30]. A worthwhile difference was determined as a moderate effect size >0.6, with the imprecision of model regression parameter estimates expressed using 95% confidence intervals (CI). Descriptive statistics of the absolute total match outputs were calculated to provide context to peak match running demands.

## Results

**Fig 1:**
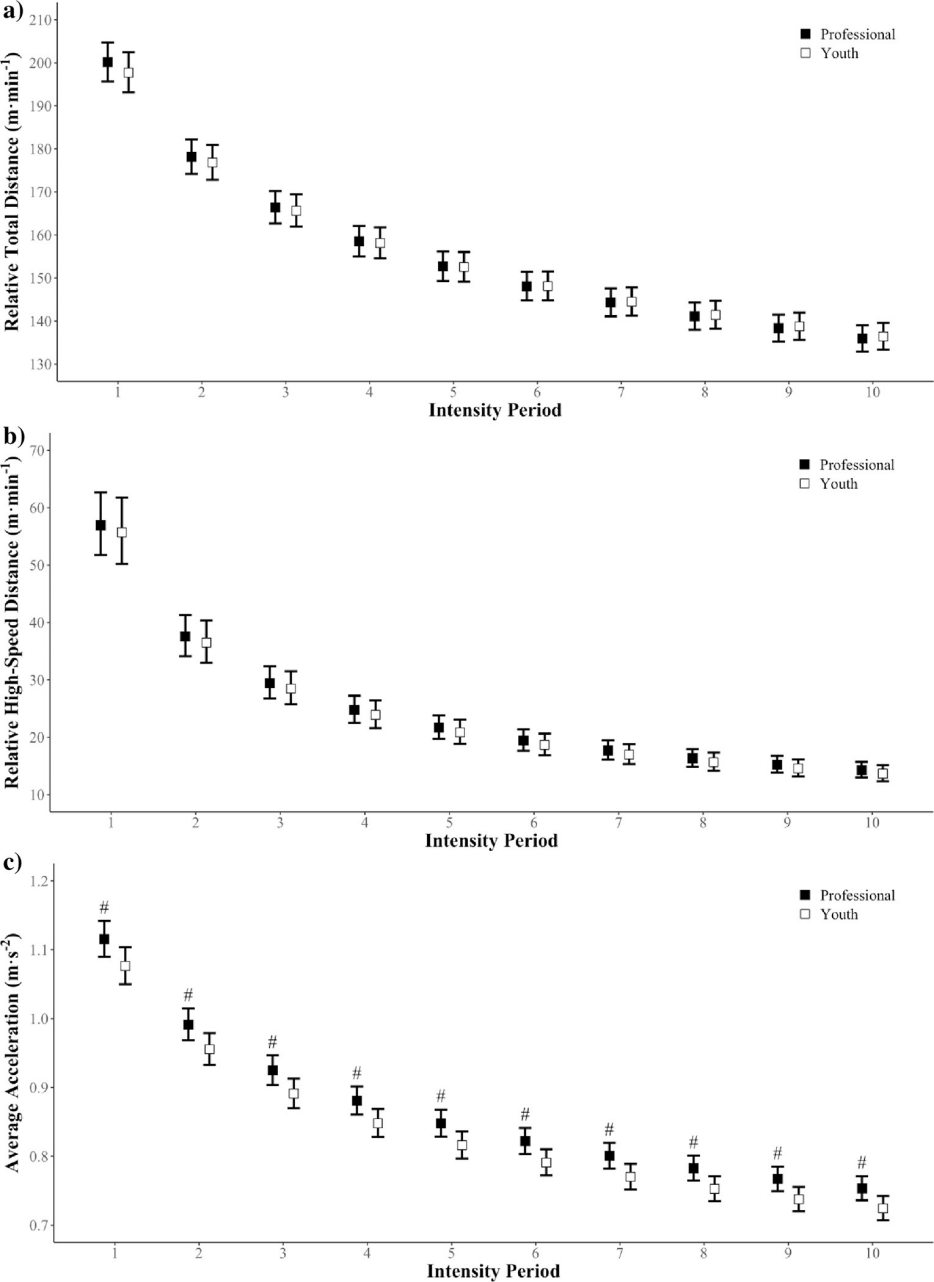
A comparison between youth and professional competitions for peak match running intensities of a) relative total distance, b) relative high-speed distance and c) average acceleration. ^#^ = moderate difference to youth (ES = 0.6–1.2).

The total physical outputs were similar between the elite youth and professional competition levels for all metrics of running performance (see Table 1 below). Differences in peak TD demands between competitions for epochs of 2–10 min were *trivial* (SMD = 0.01–0.15) with only a *small* difference (SMD = 0.25) observed for the 1 min epoch (see Fig 1A). Similarly, peak HSD (Fig 1B) demands showed *trivial* differences (SMD = 0.09–0.18) between competition levels across all epochs. However, the peak AveAcc demands (Fig 1C) were *moderately* greater (SMD = 0.63–0.69) for the professional than the youth competition for all epoch durations. Intercept and exponent values of the non-linear power law models are presented in Table 2.

**Table 1:** Absolute and relative physical output total match averages. Data presented as mean ± SD. Data in brackets indicate relative distances covered (m·min^-1^). Match duration is inclusive of added time.

Competition	Match Duration (min)	Total Distance (m)	High-Speed Distance (m)	Average Acceleration (m·s^-2^)
Professional	97 ± 3	11, 035 ± 698(114 ± 8)	725 ± 250(8 ± 3)	0.64 ± 0.05
Youth	97 ± 2	11, 333 ± 1048(116 ± 11)	713 ± 289(7 ± 3)	0.64 ± 0.05

No differences observed between competition levels.

**Table 2:** Non-linear relationships of power law models by level of competition. Data are presented as means, with the 90% CI presented in brackets.

Competition	Total Distance		High-Speed Distance		Average Acceleration	
	Intercept	Slope	Intercept	Slope	Intercept	Slope
Professional	200(195–205)	-0.168(-0.170, -0.166)	57(51–63)	-0.600(-0.608, -0.592)	1.12(1.09–1.14)	-0.170(-0.173, -0.168)
Youth	198(193–203)	-0.161(-0.166, -0.157)	56(50–62)	-0.610(-0.627, -0.593)	1.08(1.05–1.10)	-0.172(-0.177, -0.167)

## Discussion

The current study provides insight into the total match demands and peak match running demands between youth and professional football players within a single club that employed similar tactical formations and tactical approaches. Total match running demands were similar between competition levels for all three running demand metrics. Similarly, peak match running demands of TD and HSD were similar between competition levels, with only the peak AveAcc demands being lower in the youth competition across all epoch durations. While the underlying mechanisms for this discrepancy are likely multi-factorial, it suggests that youth players need more exposure to greater magnitude of acceleration demands in training to prepare for professional competition. Further, the power law parameters provided in Table 2 can be used to design and implement training drills that develop youth players acceleration and repeat effort capacities in order to expose such players to match intensities reflective of a professional competition [4].

When preparing youth players for the rigors of professional football, a key outcome is to ensure youth players are capable of the physical outputs required of professional competitions. In the present study, players in the youth and professional competitions completed similar TD (11,035 ± 698 m vs 11,333 ± 1048 m, respectively) and HSD (713 ± 289 m vs 725 ± 250 m, respectively) volumes across a match. These total match demands are comparable to those previously reported in professional football across various international leagues [5–7, 14, 31]. Furthermore, the observed AveAcc across a match was similar between the youth (0.64 ± 0.05 m·s^-2^) and professional (0.64 ± 0.05 m·s^-2^) players, which is important as the AveAcc provides a good indicator for the overall intensity of the physical stimulus [4]. Therefore, while the technical and tactical capacities of youth and professional football players likely differ, the total match demands of youth football competition are similar to those of professional competition. This suggests that the Australian youth football competition provides sufficient physical volumes to replicate the demands of professional match play.

While quantifying the total match outputs allows the comparison of running volume between competition levels, the ability to translate these data into training drills aimed at preparing youth players may be limited due to the different tactical and technical demands. The current peak relative TD and HSD running demands were similar to previously reported data for separate elite youth and professional competitions [4, 16, 17]. However, in the current study, the peak AveAcc demands were moderately higher in the professional competition compared to the youth competition, suggesting that professional players perform more frequent or higher magnitude changes in velocity. As both acceleration and deceleration actions impose a higher metabolic cost than constant velocity running [5], youth players may not be physically prepared for the increased peak AveAcc demands of professional football, possibly due to a lower training age or a lack of exposure to the required demands. Moreover, in the Italian Serie A, more successful (league ranking) teams complete more technical involvements with the ball, (e.g. passes, tackles, shots) during match-play than less successful teams [32]. Comparatively, as the youth competition is a lower-level competition, it may require players to have fewer technical involvements than professional players. Further, the ability of youth players to recognize match situations and appropriately re-position themselves may also be inferior in comparison to professional players [33]. As such, the physical demands required for players to reposition themselves in both attacking and defensive situations may be greater in the youth competition. Additionally, the lower peak AveAcc demands observed for the youth competition may in part, be explained by the age and maturation status of the players. Age has shown to be a determining factor in a player’s ability to accelerate, both maximally and repeatedly, with older youth players (U18) shown to have better acceleration capabilities than younger youth players (<U16) [34, 35]. While the underlying mechanism for discrepancies in peak AveAcc demands between competitions is likely multi-faceted, it is inferred that youth players must develop their ability to continually change velocities through tailored training stimuli that progressively replicates the demands of professional match play.

While it is acknowledged that the data in this study have been collected from only one team at each competition level, it is important to recognise that both teams were from the same club which the formation, tactical approaches and playing philosophy of the youth team mirroring that of the professional team. Contrary to previous literature which has been limited to inferring differences between competitions, the current study provides the first direct comparison between the physical demands of youth and professional football competition. One limitation is that the present study was unable to assess any internal measures, with it possible that, despite physical demands being largely similar between competitional levels, one group may have had a higher physiological cost of performing such demands. As such, investigations into the peak physiological demands of football competition and discrepancies between competition levels is warranted. Further, it is also acknowledged that in the present study, all positional groups were amalgamated, and although this was partially controlled for through the use of the same formation and tactical approaches, further investigation into positional differences between competition levels is warranted. While discrepancies in peak physical demands have been reported, the distribution of physical, technical, and tactical demands represented within each discrete time epoch requires further elucidation. For example, as peak match epoch length increases, other team sports have identified an increased frequency of technical involvements, with a resultant decrease in movement demands [36]. Future studies should aim to quantify the technical and tactical demands of these peak running periods in football to provide a greater insight to the holistic demands of the most physically demanding passages of match-play and further identify discrepancies between youth and professional competitions.

Understanding the discrepancy between the physical demands of youth and professional football competitions provides context in developing youth footballers. While coaches of youth teams often implement similar training drills and stimuli to professional teams, the current data suggests that an extra focus may need to be placed on the development of some physical capacities. In the present study, the football players were exposed to similar peak running demands of TD and HSD as their professional counterparts, with the exception of AveAcc. Hence, as the demands of the youth match-play fail to fully reflect those of professional match play, these capacities must be developed through carefully prescribed and monitored training practices. The initial evaluation of current training practices is crucial in understanding the stimulus which a player receives during training. From this, small adjustments to already implemented drills, i.e. number of players, pitch size, drill constraints, may allow for a more appropriate stimuli that replicates match demands [37]. With running during match-play occurring in tandem with technical and tactical demands, it is important to consider the balance of physical demands with that of technical and tactical demands during training to ensure players are holistically developed [38].

Ensuring that players are capable of the increased physical demands associated with professional football training and competition is crucial in the long-term success of youth footballers. As such, progressively exposing youth players to match demands that replicate those of professional competitions is crucial. Importantly, there were no differences in the absolute and peak relative TD and HSD covered between competitions, although the peak AveAcc demands were moderately lower in the youth football players. As such, the physical demands of youth football competition appear to largely replicate those of professional football competition within Australia, with only AveAcc differing between competitions. While the discrepancy in peak AveAcc demands of youth and professional football competitions is likely to be multi-factorial, it is evident that the youth competition did not fully replicate the physical demands of professional football. As such, training sessions provide the best opportunity to develop this physical stimulus with the evaluation and careful design and implementation of drills replicating peak average acceleration demands of professional competition crucial.

## Supporting information

S1 Raw data (XLSX)
